# MEDUSA: Multi-Scale Encoder-Decoder Self-Attention Deep Neural Network Architecture for Medical Image Analysis

**DOI:** 10.3389/fmed.2021.821120

**Published:** 2022-02-15

**Authors:** Hossein Aboutalebi, Maya Pavlova, Hayden Gunraj, Mohammad Javad Shafiee, Ali Sabri, Amer Alaref, Alexander Wong

**Affiliations:** ^1^Department of Computer Science, University of Waterloo, Waterloo, ON, Canada; ^2^Waterloo AI Institute, University of Waterloo, Waterloo, ON, Canada; ^3^Department of Systems Design Engineering, University of Waterloo, Waterloo, ON, Canada; ^4^Department of Radiology, Niagara Health, McMaster University, Hamilton, ON, Canada; ^5^Department of Diagnostic Imaging, Northern Ontario School of Medicine, Thunder Bay, ON, Canada; ^6^Department of Diagnostic Radiology, Thunder Bay Regional Health Sciences Centre, Thunder Bay, ON, Canada

**Keywords:** computer vision, deep neural net, COVID-19, chest X-ray (CXR), diagnosis

## Abstract

Medical image analysis continues to hold interesting challenges given the subtle characteristics of certain diseases and the significant overlap in appearance between diseases. In this study, we explore the concept of self-attention for tackling such subtleties in and between diseases. To this end, we introduce, a multi-scale encoder-decoder self-attention (MEDUSA) mechanism tailored for medical image analysis. While self-attention deep convolutional neural network architectures in existing literature center around the notion of multiple isolated lightweight attention mechanisms with limited individual capacities being incorporated at different points in the network architecture, MEDUSA takes a significant departure from this notion by possessing a single, unified self-attention mechanism with significantly higher capacity with multiple attention heads feeding into different scales in the network architecture. To the best of the authors' knowledge, this is the first “single body, multi-scale heads” realization of self-attention and enables explicit global context among selective attention at different levels of representational abstractions while still enabling differing local attention context at individual levels of abstractions. With MEDUSA, we obtain state-of-the-art performance on multiple challenging medical image analysis benchmarks including COVIDx, Radiological Society of North America (RSNA) RICORD, and RSNA Pneumonia Challenge when compared to previous work. Our MEDUSA model is publicly available.

## 1. Introduction

The importance of medical imaging in modern healthcare has significantly increased in the past few decades and has now become integral to many different areas of the clinical workflow, ranging from screening and triaging, to diagnosis and prognosis, to treatment planning and surgical intervention. Despite the tremendous advances in medical imaging technology, an ongoing challenge faced is the scarcity of expert radiologists and the difficulties in human image interpretation that result in high inter-observer and intra-observer variability. As a result, and due to advances in deep learning (1–3) and especially convolutional neural networks (4–7), there has been significant research focused on computer aided medical image analysis to streamline the clinical imaging workflow and support clinicians and radiologists to interpret medical imaging data more efficiently, more consistently, and more accurately.

For example, in the area of lung related complications, deep neural networks have been explored to great effect for aiding clinicians in the detection of tuberculosis (8, 9), pulmonary fibrosis (10, 11), and lung cancer (12–14). Similar works have been done for prostate cancer (15, 15, 16) and breast cancer (17, 18).

From a machine learning perspective, the area of medical image analysis continues to hold some very interesting challenges that are yet to be solved by the research community. There are two particularly interesting challenges worth deeper exploration when tackling the challenge of medical image analysis. First, certain diseases have very subtle characteristics particularly at the early stages of disease. For example, in the case of infection due to severe acute respiratory syndrome coronavirus 2 (SARS-CoV-2) virus, which is the cause of the ongoing COVID-19 pandemic, the signs of lung infections often manifests itself at the earlier stage as faint opacities in the mid and lower lung lobes that can be difficult to characterize and distinguish from normal conditions. Second, the visual characteristics of the certain disease have high intra-disease variance, as well as low inter-disease variance that makes it challenging to distinguish between diseases or characterize a given disease. For example, many of the visual characteristics for SARS-CoV-2 infections identified in clinical literature (19–24) such as ground-glass opacities and bilateral abnormalities can not only vary significantly from patient to patient and at different stages of the disease but also present in other diseases such as lung infections due to bacteria and other non-SARS-CoV-2 viruses.

An interesting area to explore for tackling these two challenges found in medical image analysis in the realm of deep learning is the concept of attention (25–27). Inspired by the notion of selective attention in human cognition where irrelevant aspects of sensory stimuli from the complex environment are tuned out in favor of focusing on specific important elements of interest to facilitate efficient perception and understanding, the concept of attention was first introduced in deep learning by Bahdanau et al. (27) for the application of machine translation. The success of attention in deep learning has led to considerable breakthroughs, with the most recent being the introduction of transformers (25, 28). Attention mechanisms in deep learning have now seen proliferation beyond natural language processing into the realms of audio perception and visual perception (29–33).

Much of seminal literature in the realm of attention for visual perception is the introduction of self-attention mechanisms within a deep convolutional neural network architecture to better capture long-range spatial and channel dependencies in visual data (32, 34–36). Among the first to incorporate attention into convolutional architectures is Hu et al. (36), who introduced channel-wise attention through lightweight gating mechanisms known as squeeze-excite modules at different stages of a convolutional neural network architecture. Woo et al. (32) extended upon this notion of light-weight gating mechanisms for self-attention through the introduction of an additional pooling-based spatial attention module which, in conjunction with the channel-wise attention module, enabled improved representational capabilities and state-of-the-art accuracy.

More recently, there has been a greater exploration of stand-alone attention mechanisms used both as a replacement or in conjunction with convolutional primitives for visual perception. Ramachandran et al. (35) introduced a stand-alone self-attention primitive for directly replacing spatial convolutional primitives. Hu et al. (37) introduced a novel local relation primitive which utilizes composability of local pixel pairs to construct an adaptive aggregation weights as a replacement for convolutional primitives. Wu et al. (33) and Dosovitskiy et al. (38) both studied the direct utilization of transformer-based architectures for visual perception by tokenizing the input visual data.

A commonality between existing attention mechanisms in the research literature is that selective attention is largely decoupled from a hierarchical perspective, where lightweight attention mechanisms with limited individual capacities act independently at different levels of representational abstraction. As such, there is no direct global attentional context between scales nor long-range attentional interactions within a network architecture. Our hypothesis is that the introduction of explicit global context among selective attention at different levels of representational abstractions throughout the network architecture while still enabling differing local attention context at individual levels of abstractions can lead to improved selective attention and performance. Such global context from a hierarchical perspective can be particularly beneficial in medical image analysis for focusing attention on the subtle patterns pertaining to disease that often manifests unique multi-scale characteristics.

To test this hypothesis, we introduce **M**ulti-scale **E**ncoder-**D**ecoder **S**elf-**A**ttention (MEDUSA), a self-attention mechanism tailored for medical image analysis. MEDUSA takes a significant departure from existing attention mechanisms by possessing a single, unified self-attention mechanism with higher capacity and multiple heads feeding into different scales in the network architecture. To the best of the authors' knowledge, this is the first “single body, multi-scale heads” realization of self-attention where there is an explicit link between global and local attention at different scales.

The paper is organized as follows. First, the underlying theory behind the proposed MEDUSA self-attention mechanism is explained in detail in section 2. The experimental results on different challenging medical image analysis benchmarks are presented in section 3. A discussion on the experimental results along with ablation studies are presented in section 4. Conclusions are drawn and future directions are discussed in section 5.

## 2. Methodology

In this section, we introduce MEDUSA mechanism that explicitly exploits and links between both global attention and scale-specific local attention contexts through a “single body, multi-scale heads” realization to facilitate improved selection attention and performance. First, we present the motivation behind this design. Second, we describe the underlying theory and design of the proposed MEDUSA self-attention mechanism. Third, we present a strategy for effectively training such a mechanism.

### 2.1. Motivation

In this study, we are motivated by recent success in research literature in leveraging attention mechanisms (25, 34, 39). As explained in (25), attention provides the amplification through weight distribution of certain features in the input that has more impact on determining the output. It is also trainable which means the weight distribution can be learned to improve representational power around specific tasks and data types.

While attention mechanisms have been shown in previous studies to lead to significant improvements in representational capabilities and accuracy for visual perception, their designs have involved the integration of lightweight attention blocks with limited capacity that are learned independently in a consecutive manner. As a result, the attention blocks are largely decoupled from a hierarchical perspective, and thus there is no explicit global attention context between scales and no long-range attention interactions. This independent attention modeling can potentially attenuate the power of attention mechanisms, especially in medical imaging data with subtle discriminative disease patterns with unique multi-scale characteristics. As such, it is our hypothesis that the introduction of global attention context for explicitly modeling the interactions among selective attention at different scales alongside scale-specific local attention contexts, all learned in a unified approach can boost the representational capabilities of deep neural networks.

Motivated by that, here, we learn explicit global context among selective attention at different levels of representational abstractions throughout the network architecture. This is achieved *via* a global encoder-decoder attention sub-module from the input data directly, as well as learning different local channel-wise and spatial attention contexts tailored for individual levels of abstractions *via* lightweight convolutional attention sub-modules. These sub-modules are connected at different layers of neural network architecture, based on both global context information from the encoder-decoder and activation response information at a given level of abstraction. This “single body, multi-scale heads” realization of selective attention not only has the potential to improve representational capabilities but also results in efficient weight sharing through interconnections between scale-specific local attention contexts through the global attention context *via* the shared encoder-decoder block. This weight-sharing process allows the network to be significantly faster and allows the network to apply the same global attention map across different scales dynamically through the corresponding scale-specific attentions. As a result, the network's training can be smoother as the attention layers are all aligned and synchronized.

In the next sections, we explain how global and local attention mechanisms are formulated in a unified structure within MEDUSA.

### 2.2. Multi-Scale Encoder-Decoder Self-Attention Mechanism

As mentioned earlier, by providing two levels of attention mechanisms (global and local attention), we facilitate a deep neural network architecture that learns both macro-level and micro-level dependencies between information within an image. While the characterization of micro-level dependencies facilitates fine-grained attention within small local regions, characterization of macro-level dependencies enables the neural network to focus attention from a global relational perspective taking relationships within the entire image into account. As such, by leveraging a combination of macro-level and micro-level dependencies in unison to facilitate global and local attention, the proposed MEDUSA attention mechanism enables progressive, more guided attention as information propagates throughout the network. This is illustrated in **Figure 4**, where at first the deep neural network is able to correctly focus its attention on the lung regions in the image (thanks to macro-level dependency characterization), and as we propagate deeper in the network it is able to focus attention on specific areas in the lungs that are important in determining whether there is the presence of disease (thanks to micro-level dependency characterization).

The proposed local attentions aims to explicitly model the global attention mechanisms in a unified framework with significantly higher capacity by incorporating multiple scale-specific heads feeding into different scales of the main network architecture. This unified framework improves the modeling capacity of the self-attention module by feeding the local attentions with global long-range spatial context and enables them at different scales to improve selective attention. To this end, the global modeling is formulated by an Encoder-Decoder block feeding into scale specific modules given the input sample.

Given the input sample *x* ∈ *R*^*h*×*w*×*c*^ where *h, w, c* are the height, width, and the number of input channels, the Encoder-Decoder, G(·), models the global attended information in a new feature space ${\text{A}}_{G}$ with the same dimension as the input data and $A_{G}\in R^{h\times w\times c}$ a sample point in that space. In the next step, the vector $A_{G}$ is fed into a multiple scale-specific attention model L(·) to extract the consistent attended information for each scale specifically given the output feature map *F*_*j*_ of the scale *j* and the global attention vector $A_{G}$. The output of $L\left(A_{G},F_{j}\right)$ is the final attention map corresponding to the scale *j* combined with both global and local attention in one unique map which is then multiplied by the feature map at scale *j* before feeding into the next processing block.

**Figure 1:**
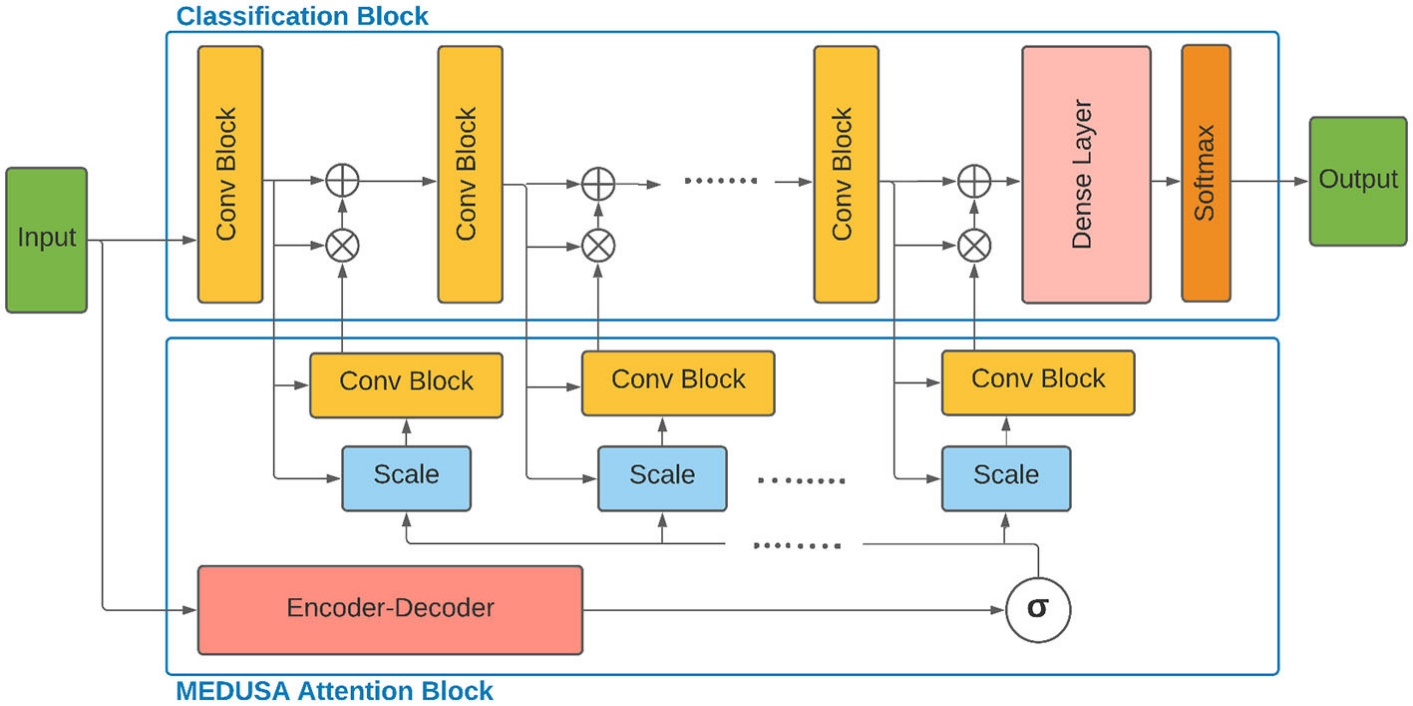
Architecture of the proposed multi-scale encoder-decoder self-attention (MEDUSA) and how it can be incorporated to a deep neural network. The global component of MEDUSA is fed by the input data which its output is connected to different scales through the network via the multi-scale specific module. The scale box here refers to the bilinear interpolation operation on the output of $\sigma \left(A_{G}\right)$ function based on the width and height of the feature map of the corresponding convolutional block. Here, we have only drew three convolutional blocks but the network can have an arbitrary number of convolutional blocks.

As seen in Figure 1, the global attention block G(·) generates a unified attention map given the input sample and acts as a synchronizer among local attention blocks and interconnect them to be synced on the most important global attention information in the training process. The two main components of MEDUSA are described as follows.

- **Global attention:** The Encoder-Decoder G(·) assimilates the global attention from the input image, with one main constraint to be consistent with different scale attentions blocks in the network. This is enforced during training when different scale attention errors are back-propagated through Encoder-Decoder G(·).
  The Encoder block takes the input *x* and maps it to a latent space feature map *z* ∈ *R*^*h*^′ × *w*′ × *c*′ with a downsampling network *D*. The latent space feature map *z* has lower dimensions compared to *x* due to the removal of non-relevant features by the downsampling network *D*. Next, given the context vector *z*, the decoder block generates the output $A_{G}$ with the upsampling network *U*. Here, it is assumed that $A_{G}\in R^{n\times m\times c}$ which provides a weight map corresponding to every pixel of the input image. One benefit of this approach is that the weight map can provide good insights (e.g., to radiologists given the medical application) to determine and illustrate how it comes to a decision. The main purpose of using the Decoder network is to provide such human readable visualization.
- **Local attention:** The global attention maps need to be transformed to scale-specific attention feature map before feeding to the main network. This task can be carried out by the multiple scale-specific attention to connecting the MEDUSA attention block properly to the classification blocks at different scales. Assume the classification block consists of *J* convolutional blocks with corresponding feature maps *F*_1_, …, *F*_*j*_, …, *F*_*J*_ where $F_{j}\in R^{n_{j}\times m_{j}\times c_{j}}$.
  Given the feature map *F*_*j*_, MEDUSA attention block infers a 3D attention map ${Ā}_{j}\in R^{h_{j}\times h_{j}\times c_{j}}$, which is applied on the feature map *F*_*j*_ to transform and generate ${\bar{F}}_{j}$. The overall process can be formulated as follow:
(1) $${\bar{F}}_{j}={ℒ}_{j}(F_{j},A_{G})⊗F_{j}+F_{j}$$
  Where ⊗ denotes element-wise multiplication and $A_{G}$ is the global attention maps generated by Encoder-Decoder G(·). To make MEDUSA an efficient operation, the scale-specific modules L(·) are formulated as follows:
(2) $${\bar{A}}_{j}=ℒ(F_{j},A_{G})=C_{j}\left({A^{\prime }}_{j},F_{j}\right)\text{ s.t. }{A^{\prime }}_{j}={ℬ}_{j}(\sigma \left(A_{G}\right))$$
  σ(·) is a Sigmoid function applied elementwise on the tensor $A_{G}$. This is followed by $B_{j}$ a bilinear interpolation operation which maps the input shape width and height to *w*_*j*_ × *h*_*j*_. $C_{j}$ is a convolutional block with the output shape (*h*_*j*_ × *w*_*j*_ × *c*_*j*_).

In our experiments, we found that using one convolution layer with filter size *c*_*j*_ is enough to get good accuracy. The main benefit of using bilinear interpolation is to keep the same global attention feature map across different scales which will be later fine tuned for different scales through multiple scale-specific attention. This way, we can interpret $\sigma \left(A_{G}\right)$ to better reflect which regions of the image the network is paying attention. As a result, the $\sigma \left(A_{G}\right)$ can be used as a visual explanation tool to further analyze the predictions made by the network.

While here we incorporate the proposed MEDUSA in a classification task, the simplicity yet effectiveness of the proposed self-attention block makes it very easy to be integrated into different deep neural network architectural for different applications. New network architectures with the proposed self-attention module can take advantage of different training tricks, given the global attention component of MEDUSA is decoupled from the main network. This benefits the model to be trained in an iterative manner between the main model and the self-attention blocks and as a result, speeds up the convergence of the whole model. Moreover, this setup facilitates the model to take advantage of any pre-trained model for the main task.

### 2.3. Training Procedure

As shown in Figure 1, the global component of the proposed self-attention block is designed to be decoupled, or initially separate from the main network and then combined using local attention components. The global component of MEDUSA generates a unique long-range spatial context given the input sample which is customized by the scale-specific module to generate scaled local attentions. This decoupling approach facilitates the use of training tricks in the training of both self-attention block and the main network. To this end, we use two main tricks to train the proposed architecture:

- **Transfer learning**: First, transfer learning was used to provide a better initialization of the global component (Encoder-Decoder) in the MEDUSA attention block. In particular, for the experiment on the CXR dataset, we used U-Net (40) as the Encoder-Decoder that was pre-trained on a large (non-COVID-19) dataset for lung region semantic segmentation. Using a pre-trained semantic segmentation to initialize the Encoder-Decoder, it helps guiding the network to pay attention to relevant pixels in the image.
- **Alternating training**: As mentioned before, the designed structure of the proposed MEDUSA can decouple the global component of the self-attention block from the main network. This benefits the model to use alternating training technique for training the attention block and the main network sequentially, as the second training trick. During each step of the alternating training, one block (the main network architecture or MEDUSA attention block) is frozen interchangeably while the other one is being trained. This way, we ensure that these two blocks learn their relatively different but related tasks concurrently. In our experiments, we found that by using this technique, not only the network converges to the best solution faster but it also makes the training less computationally expensive in other aspects and the memory consumption during the training decreases considerably. As such, we can use larger batch sizes which decreases the training time.

### 2.4. Experiment Results

#### 2.4.1. Experimental Setup

In this section, we describe the evaluated dataset and hyperparameters used for reporting the experimental results.

#### 2.4.2. CXR-2 Dataset

**Figure 2:**
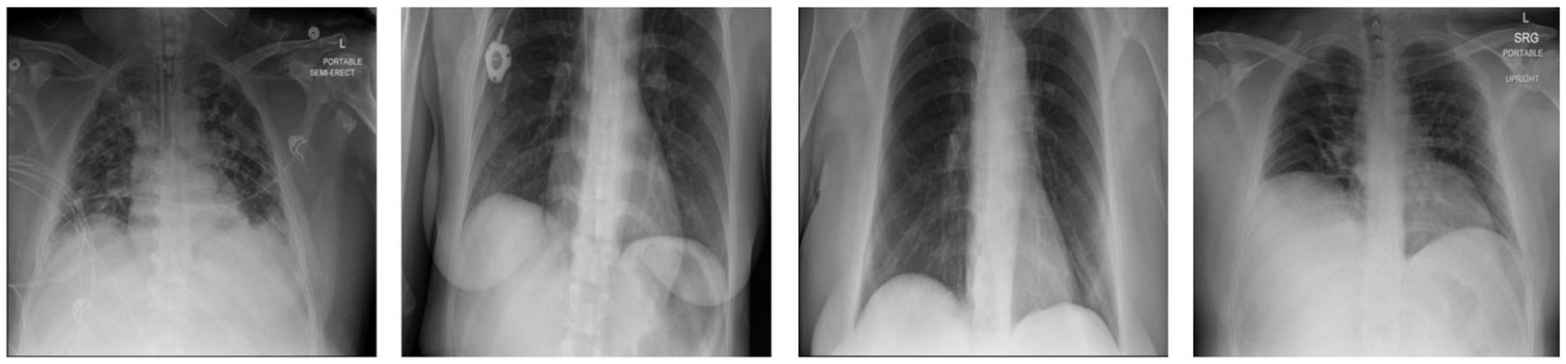
Example chest X-ray images from the benchmark dataset.

To evaluate the proposed model, it is trained on the largest CXR dataset consisting of 19,203 CXR images (41). The dataset (41) is constructed based on a cohort of 16,656 patients from at least 51 different countries. There are total of 5,210 images from 2,815 SARS-CoV-2 positive patients and the rest of images are from 13,851 SARS-CoV-2 negative patients. Interested readers can refer to Maya (41) for more information on this dataset. Figure 2 demonstrates some examples of the CXR-2 dataset.

#### 2.4.3. Architecture Design

The global component of the proposed MEDUSA is modeled by a U-Net architecture (40) which is used to provide the attention mechanism to a ResNet50 (4) architecture for a classification task. Unlike other works in the medical imaging, we did not perform any special pre-processing on the images other than resizing the images to 480 × 480. In our training, we used the Adam optimizer (42) with the learning rate of 0.00008 and the batch size of 16.

### 2.5. Results and Discussion

The evaluated results of the proposed method on the CXR-2 dataset are reported in Table 1. MEDUSA provides the highest accuracy among all the other state-of-the-art techniques with at least a margin of 2%. Moreover, compared to attentional based models including CBAM and SE-ResNet50 which utilize spatial attention and channel attention, MEDUSA outperform them by the accuracy margin of 3.55 and 13.3%, respectively. This result illustrates the importance of formulating a unified attention model in improving accuracy.

**Table 1:** Sensitivity, positive predictive value (PPV), and accuracy of the proposed network (MEDUSA) on the test data from the CXR benchmark dataset in comparison to other networks.

**Architecture**	**Sensitivity (%)**	**PPV (%)**	**Accuracy (%)**
ResNet-50 (43)	88.50	92.20	90.50
COVID-Net (44)	93.50	**100**	94.00
COVID-Net CXR-2 (41)	95.50	97.00	96.30
SE-ResNet-50 (36)	90.50	98.90	94.75
CBAM (32)	70.00	**100**	85.00
MEDUSA	**97.50**	99.00	**98.30**

*Best results are highlighted in bold*.

In addition, experimental results showed that MEDUSA and the proposed training technique lead to a much faster training convergence compared to CBAM and SE-ResNet-50 resulted in a 10X speedup in the convergence of the model. This shows that the unique design proposed for MEDUSA does not impose considerable complexity into the model's runtime cost which is a common case in other well-known attention mechanisms. Finally, as observed in Table 1, the addition of the proposed MEDUSA to the main block of the network architecture (here the ResNet-50 for classifying COVID-19) improves the accuracy by the margin of 7.8% compared to a stand alone ResNet-50, which illustrates how the proposed self-attention mechanism can help the model to better focus on important information and leads to higher performance. Table 2 shows the confusion matrix of MEDUSA. It can be observed that the proposed attention mechanism equally increases both sensitivity and specificity of the classification model.

**Table 2:** Confusion matrix of the proposed network (MEDUSA).

**SARS-CoV-2**	**Negative**	**Positive**
Negative	198	2
Positive	5	195

#### 2.5.1. Global Attention

**Figure 3:**
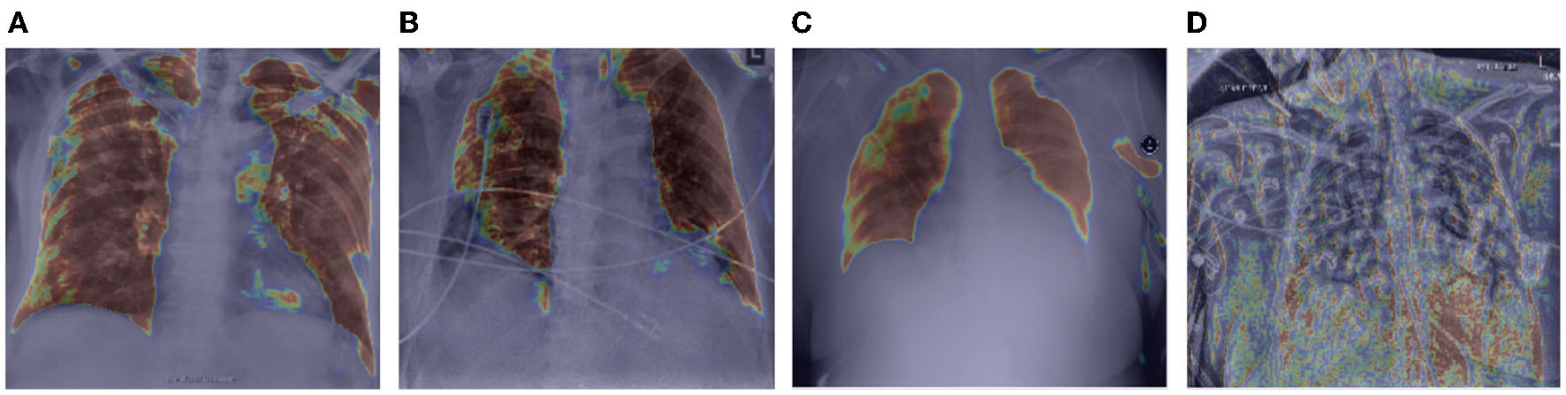
Example chest X-ray images **(A–D)** from the COVIDx CXR-2 benchmark dataset overlaid by the global attention output from the proposed MEDUSA network. The red regions indicate higher attention while blue regions indicate lower global attention.

Let us now study the behavior of the global attention sub-module of the proposed MEDUSA self-attention mechanism by visualizing its attention outputs for a variety of image examples. Figure 3 shows the global attention outputs (i.e., the output after the $\sigma \left(A_{G}\right)$ function) overlaid on the input images in the form of heat maps. The red area indicates higher global attention while blue areas indicates lower global attention. The images used here are from the same CXR-2 dataset. The model correctly classifies the cases in Figures 3A–C while the case in Figure 3D are incorrectly classified by the network.

It can be observed that while global attention is clearly focused on the lung region in the correctly classified cases in Figures 3A–C, the global attention is not focusing on the lung region in the incorrectly classified case of Figure 3D, which could be attributed to the poor quality of the chest CXR image compared to the correctly classified cases. This heat map visualization can help with better determining if the model is leveraging relevant information to infer the correct prediction which is very important in critical decision-making such as medical applications. In addition, it is interesting to notice that in the case of Figure 3B, the global attention mechanism focuses away from the wires on the chest, and thus proves to be useful for avoiding attention on unrelated, non-discriminative patterns that may otherwise be leveraged for making the right decisions for the wrong reasons. Also, we can see that in Figures 3A,C, MEDUSA helps significantly in focusing attention on the lung region for improved guidance toward relevant patterns. We believe providing this kind of visualization can greatly enhance the trust in deep learning models especially in medical applications where the decision-making causes vital outcomes.

#### 2.5.2. Local Attention

**Figure 4:**
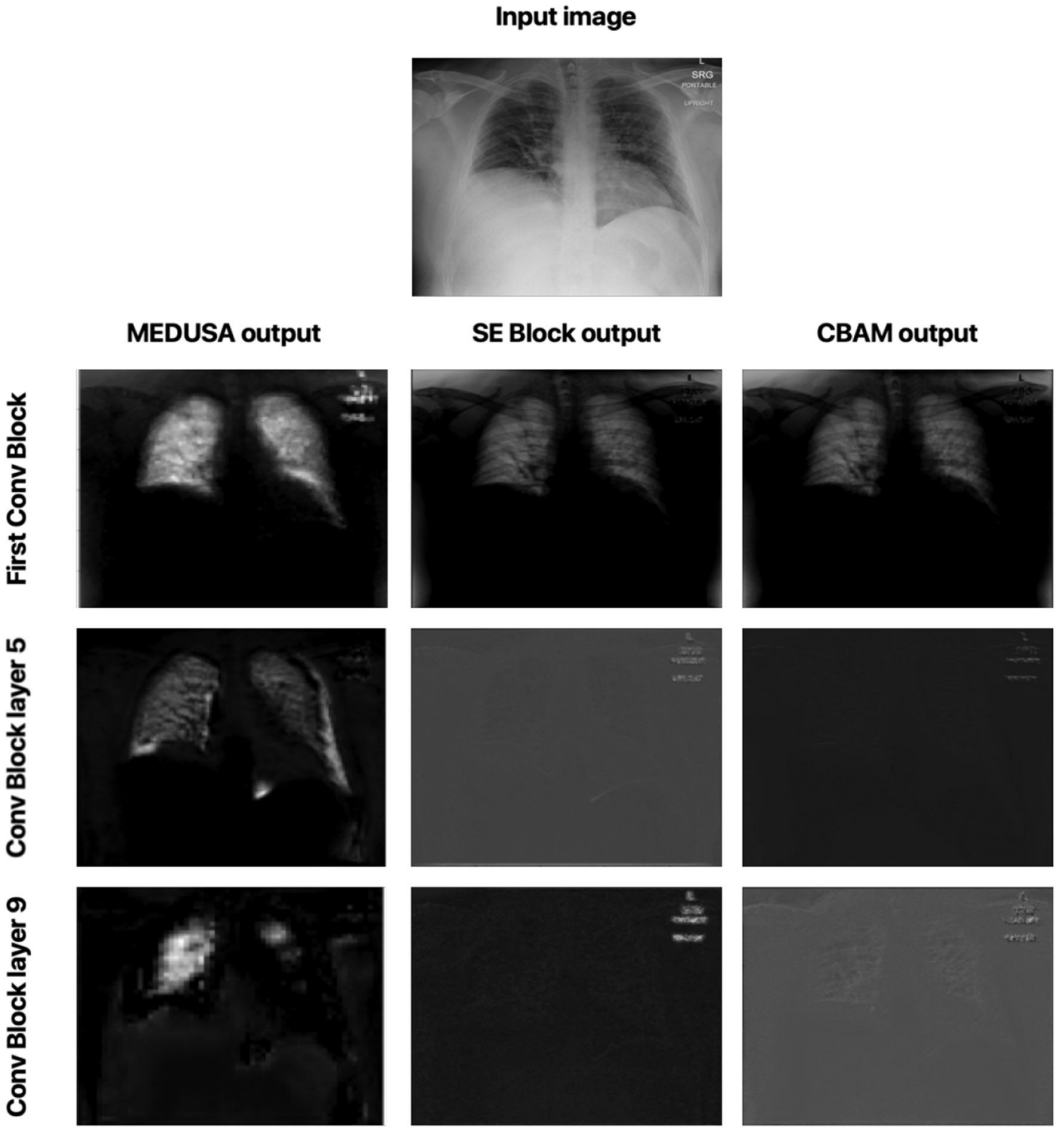
Comparison between attention-enforced outputs at different convolutional blocks when using CBAM, SE Block, and MEDUSA self-attention mechanisms. Each row demonstrates the results of the attention mechanisms on a different layer of the ResNet-50 network architecture.

Let us now study the impact of the scale-specific attention sub-modules of MEDUSA toward local selective attention. Here, we compared the activation outputs of convolutional layers at different stages of the network after asserting selective attention *via* MEDUSA, SE, and CBAM attention mechanisms. More specifically, we study the attention-enforced outputs of three different convolutional blocks (shown in Figure 4) to observe how the activation behavior evolves at different stages of the network. The whiter pixels refer to higher attention-enforced activations, with all activations normalized for visualization purposes. As seen in Figure 4, while all tested attention mechanisms can guide attention toward relevant areas of the image (e.g., lungs) in the first convolution block, as we go to the deeper blocks, the SE and CBAM mechanisms start to lose focus on these relevant areas. On the other hand, we can observe from the attention-enforced outputs where MEDUSA is leveraged that as we go deeper into the network, the attention-enforced activation outputs consistently focuses on the relevant areas for decision making while narrowing down focus toward more localized discriminative patterns within the broader area of interest in earlier blocks. This figure depicts the effectiveness of introducing global context alongside tailored local attention contexts at different scales, which provides a better hierarchical representation of the input image and the model can better extract higher level features that are more localized around the important pixels.

### 2.6. Ablation Study

In this section, we further study the impact of MEDUSA attention block to investigate to what extent it leads the network to improve its performance. Here, we specifically consider two different scenarios.

The first scenario is to see what happens if we turn off the MEDUSA attention block on ResNet-50 after the training is done. This will show us how much impact the attention block has during the training and testing as we can compare the obtained results with the base ResNet-50 model. In the second scenario, we answer the question of whether MEDUSA can only help the model to just ignore unnecessary information of the input sample or it provides scale specific attentional context through the network. To this end, the segmented result of the lung area given the input image is fed into the ResNet-50 at a singular input-head without the addition of other attention heads. In this case, we study two types of image segmentation. In the first type, we provide the CXR image which only contains the lung region, *s*(*x*) to the network, where function *s* is the segmentation operation and *x* is the unsegmented CXR image. In the second type of segmentation, we provide *x* + *s*(*x*) as the input to the ResNet-50.

The results are grouped together in Table 3. As shown in Table 3, when MEDUSA is disabled at test time, while the model still retains higher accuracy than ResNet-50 baseline by 3%, the accuracy drops to 5.3% compared to the when the MEDUSA block is still enabled during the testing. This shows that not only the MEDUSA attention block causes the ResNet-50 to attain higher accuracy during the training, but it also makes the model have higher accuracy during the testing. We also observe the similar pattern when we only provide the segmented image and remove the MEDUSA block in ResNet-50. In this regard, the network loses close to 2.5% accuracy on the test set. This proves that the MEDUSA attention mechanism is more than just a segmentation applied to the input image like what has been used in the papers (45, 46). The proposed self-attention mechanism provides multi-scale attention context which are learnt *via* a unified self-attention mechanism from a global context.

**Table 3:** Ablation study. Sensitivity, positive predictive value (PPV), and accuracy from the ablation study for the proposed network (MEDUSA) in comparison to other networks.

**Architecture**	**Sensitivity (%)**	**PPV (%)**	**Accuracy (%)**
ResNet-50 (43)	88.50	92.20	90.50
Seg Type 1 +ResNet-50 (43)	92.00	95.83	94.00
Seg Type 2 +ResNet-50 (43)	94.50	96.43	95.50
MEDUSA (Self-attention is disabled at test time)	**98.50**	88.70	93.00
MEDUSA	97.50	**99.00**	**98.30**

*Best results are highlighted in bold*.

## 3. Comprehensive Comparative Evaluation on Medical Image Analysis Datasets

In this section, we further validate the efficacy of the proposed MEDUSA self-attention mechanism through comprehensive experiments on two popular medical image analysis datasets comparing the performance of MEDUSA to other state-of-the-art deep convolutional neural networks, as well as other state-of-the-art self-attention mechanisms for deep convolutional neural networks. First, we conducted experiments and comparative analysis on MEDUSA and other tested state-of-the-art methods on the Radiological Society of North America (RSNA) Pneumonia Detection Challenge dataset (47) for the purpose of pneumonia patient case detection. Next, we conducted experiments and comparative analysis on MEDUSA and other tested methods on a multi-national patient cohort curated by the RSNA RICORD initiative (48) for the purpose of severity scoring of COVID-19 positive patients.

The source code of the proposed MEDUSA is available https://github.com/lindawangg/COVID-Net. All codes were implemented using TensorFlow version 1.15 in Python version 3.7.

### 3.1. RSNA Pneumonia Detection Challenge Dataset

**Figure 5:**
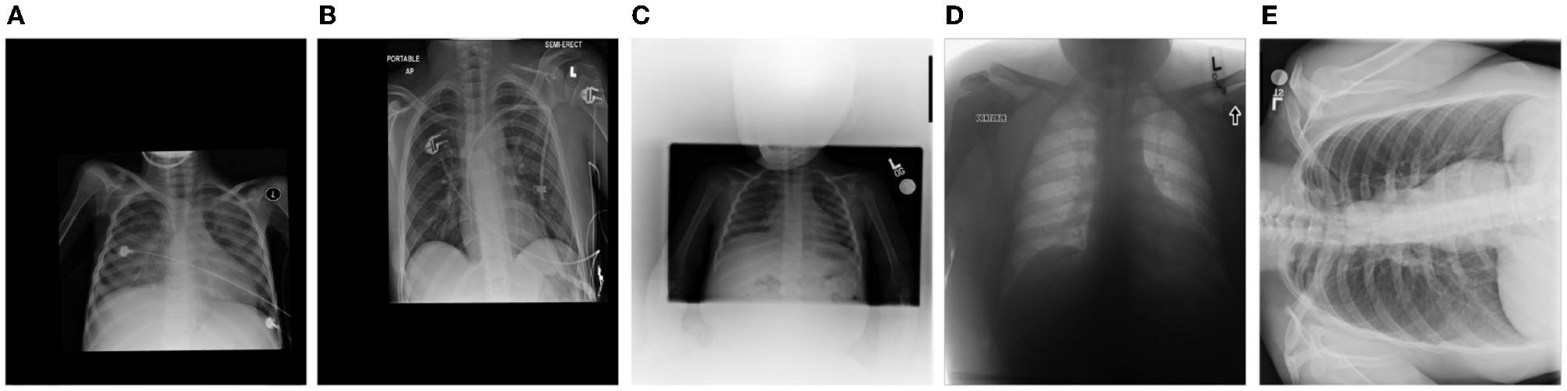
Example chest X-ray images (labeled **A–E**) with different types of significant distortions and visual anomalies from the Radiological Society of North America (RSNA) Pneumonia Challenge dataset.

This dataset was curated by the RSNA and consists of frontal-view chest X-ray images from a cohort of 26,684 patients for the purpose of Pneumonia patient case detection. The images are labeled pneumonia-positive and pneumonia-negative, with ~6,000 of which being pneumonia-positive cases. It is very important to note that, based on our deeper analysis of the data, approximately 20% of the chest X-ray images contain significant distortions and visual anomalies. Examples of such images are shown in Figure 5. Such distortions and visual anomalies make this particular dataset quite challenging and thus particularly effective for evaluating the selective attention capabilities of MEDUSA and other state-of-the-art self-attention mechanisms to focus on the right visual cues amidst such distortions.

The experimental results evaluating different networks for this dataset are depicted in Table 4. It can be observed that leveraging the proposed MEDUSA self-attention mechanism can provide significant performance improvements over other state-of-the-art self-attention mechanisms, leading to over 6% higher accuracy when compared to other methods. Furthermore, leveraging MEDUSA resulted in a 14% gain in sensitivity when compared to other methods. Nonetheless, CheXNet (49) provides a higher positive predictive value (PPV) among the tested networks at the cost of a significantly lower overall sensitivity.

**Table 4:** Sensitivity, positive predictive value (PPV), and accuracy of the proposed network (multi-scale encoder-decoder self-attention, MEDUSA) on the test data from the RSNA Pneumonia dataset in comparison to other networks.

**Architecture**	**Sensitivity (%)**	**PPV (%)**	**Accuracy (%)**
SE-ResNet-50 (36)	40.0	90.9	68.0
CheXNet (49)	50.0	**92.6**	73.0
CBAM (32)	68.0	77.3	74.0
MEDUSA	**82.0**	83.7	**83.0**

*Best results are highlighted in bold*.

**Figure 6:**
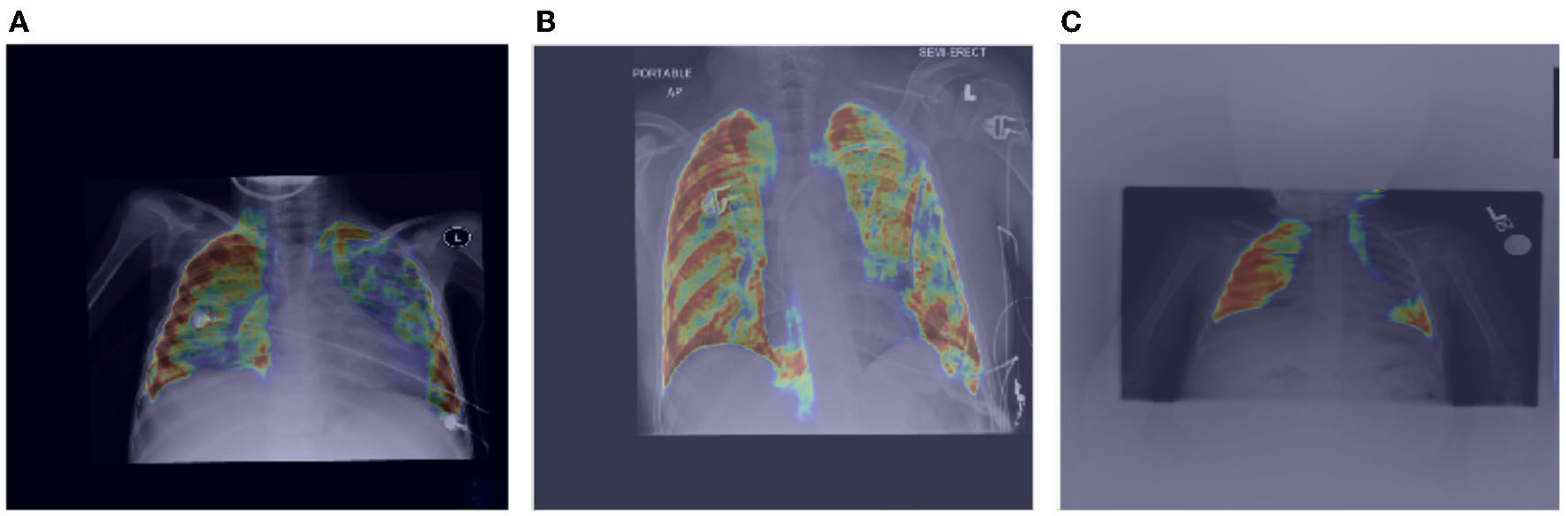
Impact of MEDUSA global attention on the images with distortion. The images **(A–C)** are corresponding to images **(A–C)** in Figure 5.

**Figure 7:**
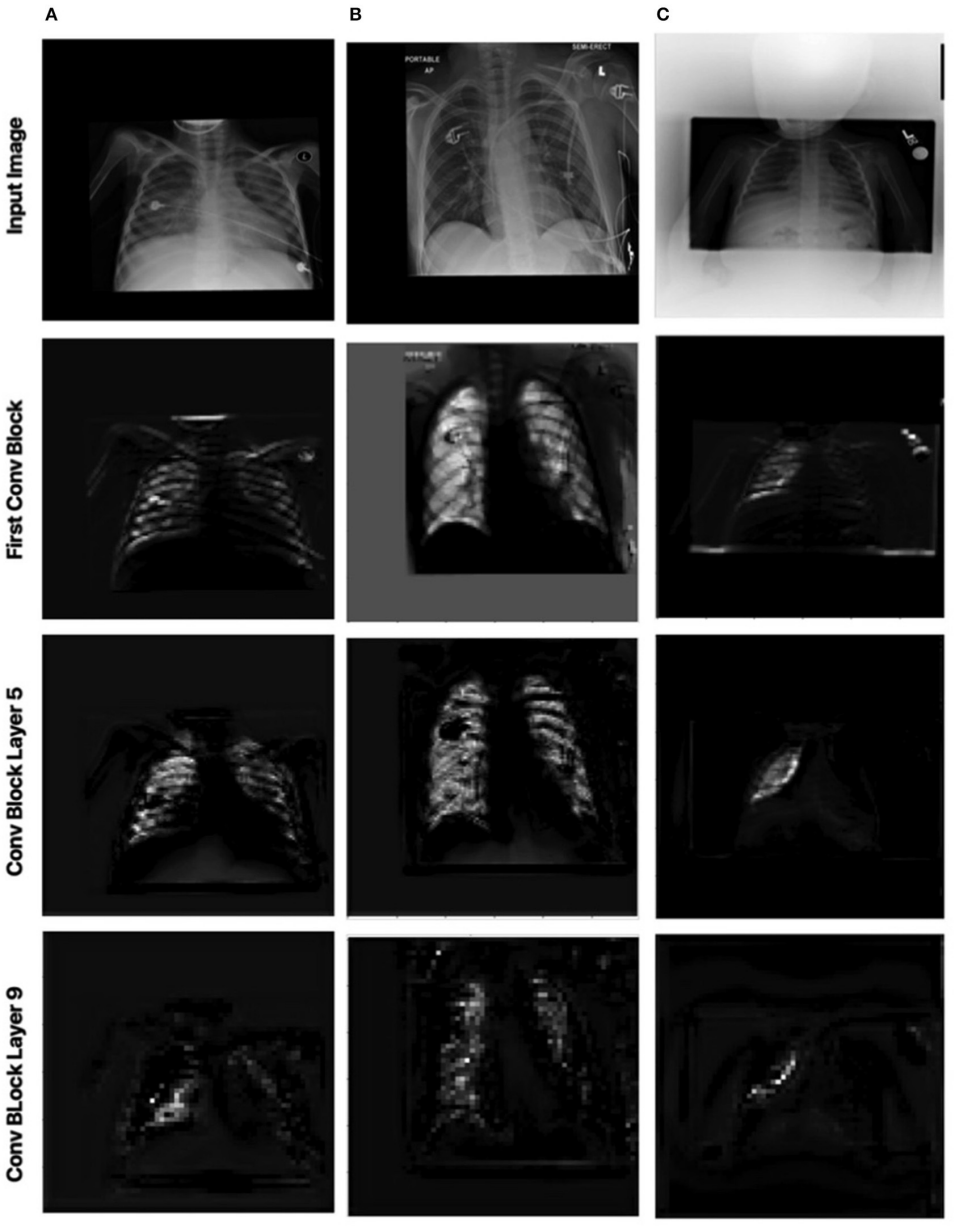
Impact of MEDUSA local attention on the images with distortion. Columns 1, 2, and 3 correspond to images **(A–C)** in Figure 5, respectively.

Here, we also investigate how MEDUSA's global and local attention impact the performance of the model when the input image is distorted. Figures 6, 7 demonstrate the global attention and local attention from MEDUSA, respectively, visualized for a subset of images shown in Figure 5. As seen, the global attention component of the proposed self-attention mechanism effectively identifies the most informative regions of the image for the model to attend to, even when the images are distorted and no additional preprocessing is applied. The proposed self-attention mechanism clearly helps the model to focus on the most important information which is confirmed by the quantitative results as well.

Figure 7 shows the local attention heads outputs which are adjusted based on the global attention map at different blocks in the convolutional network by the scale-specific modules of the proposed MEDUSA. As we go deeper into the network, the attention area is more localized on the relevant regions which are consistent with the reported results in the main paper.

### 3.2. RSNA RICORD COVID-19 Severity Dataset

**Figure 8:**
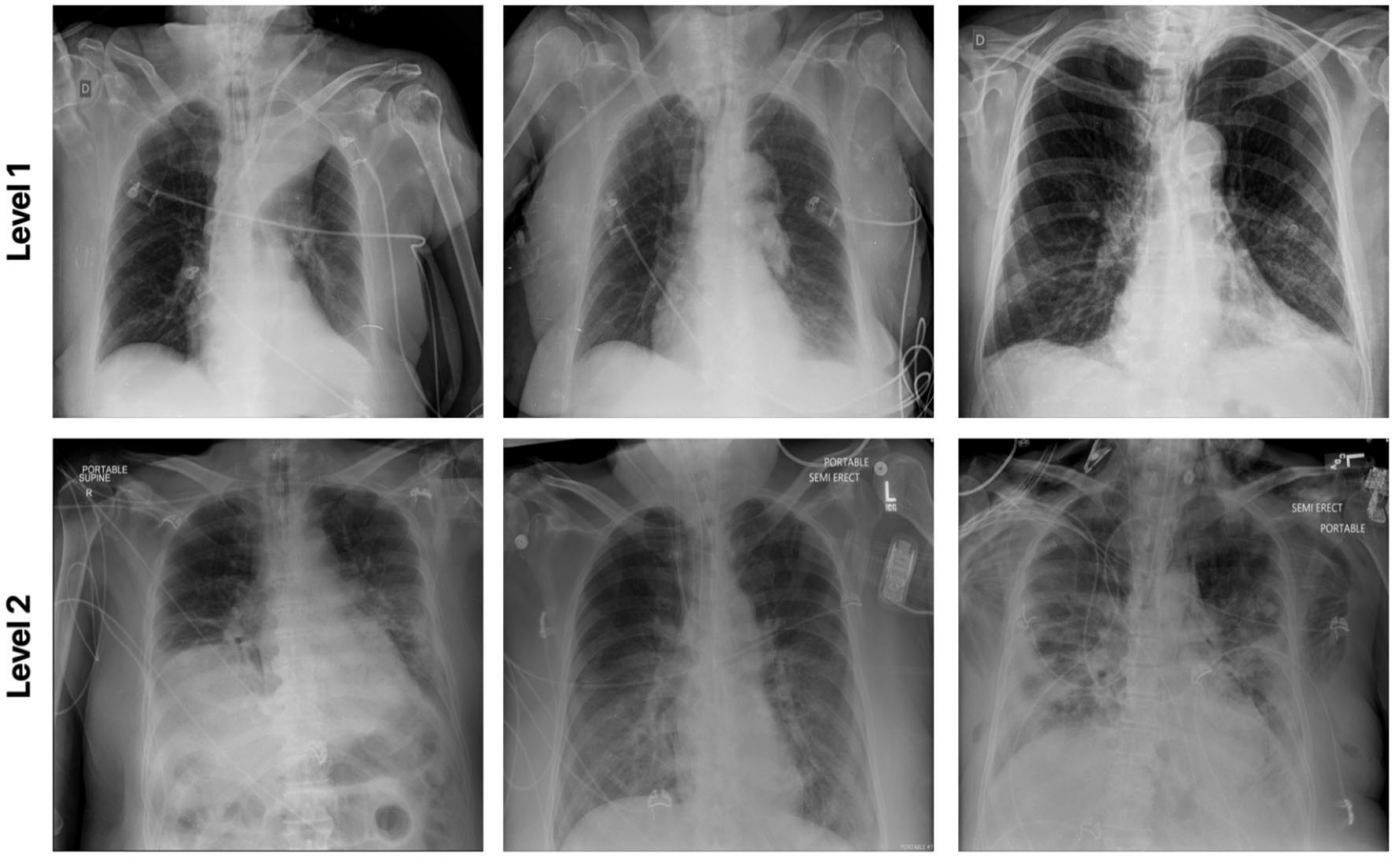
Example chest X-ray images from the RSNA RICORD COVID-19 Severity dataset.

This dataset was curated by the RSNA and consisted of chest X-ray images with full annotations on the severity condition score associated with COVID-19 positive patients. Each lung is split into 3 separate zones (a total of 6 zones for each patient) and the opacity is measured for each zone. Here for the experimental result, the patient cases are grouped into two airspace severity levels: Level 1: opacities in 1 or 2 zones and Level 2: opacities in 3 or more zones. The multi-national patient cohort in this dataset consists of 909 CXR images from 258 patients. Among the 909 CXR images, 227 images are from 129 patients with Level 1 annotation and the rest of the images are grouped with Level 2 class label. Figure 8 illustrates example CXR images from this dataset for the different airspace severity level groups.

The efficacy of the proposed MEDUSA self-attention mechanism and that of other state-of-the-art methods are shown in Table 5, with sensitivity and PPV values being reported for Level 2 patient cases. Again, we observe that MEDUSA has superior accuracy and positive predictive value (PPV) when compared to other approaches. The proposed MEDUSA self-attention mechanism provided over 8.6% higher accuracy and over 11.2% higher PPV than compared to SE and CBAM self-attention mechanisms. While leveraging the SE self-attention mechanism resulted in the highest sensitivity in this experiment, its overall accuracy is lower due to its poor performance on Level 1 patient cases.

**Table 5:** Sensitivity, positive predictive value (PPV), and accuracy of the proposed network (MEDUSA) on the test data from the RSNA RICORD COVID-19 severity dataset in comparison to other networks.

**Architecture**	**Sensitivity (%)**	**PPV (%)**	**Accuracy (%)**
SE-ResNet-50 (36)	**90.8**	77.4	76.7
CheXNet (49)	63.46	84.62	83.33
CBAM (32)	84.0	73.0	70.0
MEDUSA	85.0	**88.6**	**85.3**

*Best results are highlighted in **bold***.

## 4. Conclusion

In this paper, we proposed a novel attention mechanism *so-called* MEDUSA which is specifically tailored for medical imaging applications by providing a unified formulation for the attention mechanism. The global context is modeled explicitly among selective attention at different scales and representational abstractions throughout the network architecture which can help to model the scale-specific attention more effectively. This unified framework provides a more coherent attention mechanism at different scales to the network leading to more accurate attention context and higher performance as a direct result. Our results attest that the current model is not only faster than some of the predecessors, but is also able to achieve higher accuracy. While the results showed the effectiveness of the proposed attention mechanism on image based and medical applications, we aim to introduce the new version of the proposed MEDUSA in designing new architectures for other problems such as NLP and sequential data. Moreover, new training techniques to speed up the convergence and improve the model accuracy is another direction of the future work.

An interesting future direction is to leverage the proposed MEDUSA architecture for the purpose of CT image analysis. While the realization of the proposed MEDUSA attention mechanism currently leverages two-dimensional convolutional blocks given the nature of CXR images explored in this study, extending MEDUSA to three-dimensional convolutional blocks would enable volumetric global and local attention for volumetric medical imaging data. This would be worth a deeper exploration in the future.

Finally, while in this study, we mainly focused on chest X-ray analysis of COVID-19, our proposed MEDUSA framework has broader potential in medical image analysis beyond the studied clinical workflow tasks and modality. In this regard, MEDUSA can be used for a wide range of applications ranging from disease detection, risk stratification, and treatment planning for a wide range of diseases such as tuberculosis (8, 9), pulmonary fibrosis (10, 11), prostate cancer (50–53), breast cancer (17), and lung cancer (12–14) using different modalities ranging from ultrasound to MRI to CT to PET imaging.

## Data Availability Statement

The MEDUSA network and associated scripts are available in an open source manner at http://www.covid-net.ml, referred to as COVIDNet CXR-3. Further inquires can be directed to the corresponding author/s.

## Author Contributions

All authors listed have made a substantial, direct, and intellectual contribution to the work and approved it for publication.

## Conflict of Interest

The authors declare that the research was conducted in the absence of any commercial or financial relationships that could be construed as a potential conflict of interest.

## Publisher's Note

All claims expressed in this article are solely those of the authors and do not necessarily represent those of their affiliated organizations, or those of the publisher, the editors and the reviewers. Any product that may be evaluated in this article, or claim that may be made by its manufacturer, is not guaranteed or endorsed by the publisher.
